# Prospective video-based analysis of coronal lower limb alignment may be as accurate as radiography in children

**DOI:** 10.1186/s12891-025-09343-y

**Published:** 2025-11-27

**Authors:** L. Wystrach, M. Wendt, L. Bode, EJ Kubosch, K. Kuminack, H. Schmal, M. Wenning

**Affiliations:** 1 Department of Orthopedic and Trauma Surgery, Medical Center – Albert-Ludwigs-University of Freiburg, Freiburg, Germany; 2 Orthopedic Surgical Office Darmstadt, Darmstadt, Germany; 3 Department of Orthopedic Surgery, BDH Klinik Waldkirch, Waldkirch, Germany; 4 OrthoTeam Freiburg, Freiburg, Germany

**Keywords:** Kinematics, Knee- and hip angles, Motion‐analysis software, Guided growth, epiphysiodesis, children

## Abstract

Temporary hemiepiphysiodesis is a growth-guided surgical technique to address lower limb malalignment in children with remaining growth potential. It is a minimally invasive approach that relies on full standing radiography of the lower limb, resulting in exposure to ionizing radiation. Radiation-free, video-based alternative techniques have recently evolved which seem to have potential to be applied in clinical pathways, but they are still very expensive and have a complex setup.

In this prospective pilot study, a low budget and uncomplicated method to assess malalignment of the lower limb was compared to radiologic measurements in 40 children aged from 8 to 16 years with idiopathic genu varum or valgum. Dynamic and static video-based assessments of the children were conducted, during their routine visits provided they had undergone full standing radiographs of the lower limb. Measurements of the primary (HKA = hip-knee angle) and secondary knee angles (e.g. medial proximal tibia angle) were compared. It was observed that the video-based hip-knee-ankle angle correlated very closely with radiographic values, as well as a significant positive correlation (*p* < 0.001) between all techniques (radiographic/dynamic/static). Moreover, the HKA measurements revealed excellent to high inter- and intrarater reliability.

Concerning the HKA, this low-budget technique may represent a reliable alternative for longitudinal evaluation of lower limb malalignment in children. Considering the measurement errors in video-based analyses, it may be suggested that radiographic follow-up is only indicated until the target HKA approximates 2°. In summary, this novel technique has the potential to be the first step towards implementing radiation-free setups within clinical routine to evaluate lower limb malalignment. Future studies will need to determine whether the reliability and quality of video-based techniques are high enough to guide operative decision making.

**Supplementary Information:**

The online version contains supplementary material available at 10.1186/s12891-025-09343-y.

## Introduction/background

Lower limb malalignment is one of the main reasons for orthopedic consultations in children [1]. A deviation in the lower limb axis occurs as the limbs develop physiologically. At birth, the physiological lower limb alignment is characterized by a varus configuration of approximately 15°, which is primarily located in the tibia. By the time the child begins to walk, the knee should have reached a neutral alignment. During further development, a valgus alignment emerges, peaking at around 10° between the ages of 3 and 4 years. Thereafter, the valgus gradually decreases until the age of approximately 10 years, when the physiological valgus of 5–7° typical of adults is attained. At this stage, both the femoral condyles and the malleoli are in contact, which is perceived as a “straight” leg axis. In some patients, pathologic malalignment aside from the natural history develops or becomes even worse after the age of eight [2–4]. This malalignment can originate from a traumatic, metabolic, or syndromic condition, but in most patients it is an idiopathic disorder [1]. As a rule, those who develop a persistent malalignment need to be monitored closely. There is currently no established non-operative intervention that prevents progression of the malalignment or even induces improvement. In the last few decades, a surgical technique for minimally invasive correction, namely temporary hemiepiphyseodesis, has evolved. It utilizes the remaining growth potential of the epiphysis as a means to correct malalignment [5, 6]. The use of small implants bridging the epiphysis on one side (hemiepiphyseodesis) leads to a temporary, asymmetric arrest of activity, enabling corrective, compensatory growth on the contralateral side.

Indications for surgical intervention include an increased risk of unilateral osteoarthritis due to asymmetric weight bearing, marked leg length discrepancies, and, in some cases, cosmetic concerns [7–9]. The static full-length standing radiograph is still the gold standard for diagnosing coronal malalignment of the lower limbs. Such radiographs can be obtained in pediatric populations at initial consultation when a severe malalignment is suspected. If a malalignment is confirmed, radiographs are indicated depending on the treatment follow-up. If surgical therapy via hemiepiphysiodesis is performed, follow-up radiographs are mandatory to define the optimal point of implant removal to avoid severe overcorrection. Young children are exposed to ionizing radiation while undergoing repeated radiography. Exact values for the effective radiation dose of standing full-length radiographs in children are not consistently reported in literature. However, since the pelvis is included in the field of view, the dose must be higher than that of a pelvic radiograph, which was measured at less than 0.07 mSv effective dose in Australian children [10]. For comparison, this is roughly equivalent to the cosmic radiation received during about 29 h of air travel. Furthermore, dynamic forces during movement cannot be visualized by this static diagnostic tool, meaning that pathologic weight bearing in the knee compartments during movement is not assessable at all, so far we can only analyze anatomical and mechanical alignment in radiographs [11–13].

Recent technical innovations has made it possible to measure the static and dynamic leg axis via video analysis. Moreover, it enables us to assess dynamic characteristics and compensatory effects on the gait using a radiation-free alternative to standard radiographs [14–17]. Several published studies have described how this methodology was applied in adult patients using a variety of videographic approaches, motion capture systems and analysis tools [18]. However, when it comes to avoiding ionizing radiation, it is pediatric populations that would benefit most from minimizing this burden. The use of such novel techniques in a pediatric setting, therefore has great potential provided they are methodologically reliable. To date, no study has focused on this technique’s methodological accuracy in a pediatric population.

The aim of this study was therefore to analyze the diagnostic accuracy and reliability of video-based lower limb alignment findings compared to the current gold-standard conventional radiographic findings. This may reduce the cumulative dose of ionizing X-rays in a pediatric population. We chose to use a technical setup that could be easily implemented in clinical routine and is available at low cost.

## Methods

This cross-sectional, prospective single-cohort study was performed according to the Declaration of Helsinki in its current version. All participants and their parents provided informed consent prior to participation. The study was approved by the local ethics committee (ETKFR #21–1319) and it was registered at the German Registry of Clinical Studies (DRKS00026070).

### Participants

**Fig. 1:**
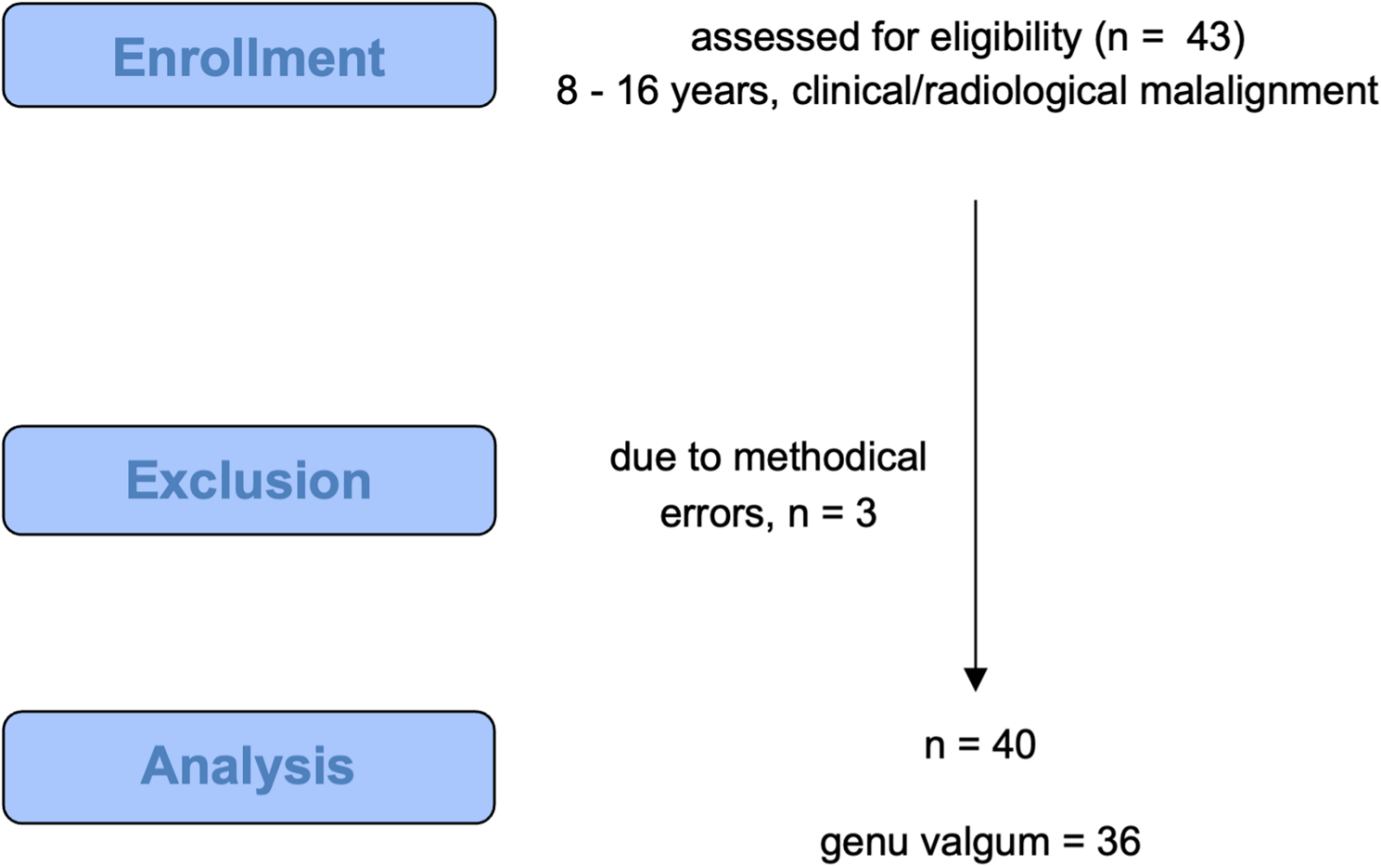
Flow chart of patient recruitment and analysis

All participants were recruited from an outpatient clinic between December 2021 and January 2023. Inclusion criteria were children and adolescents between 8 and 16 years of age with no other comorbidities other than clinical genu valgum or varum. The indication for a full-length standing radiograph of the lower limb in anteroposterior orientation was based upon clinical decision-making and was not part of the study. To form a homogeneous study cohort, our exclusion criteria were any neuromuscular disorders, other types of skeletal dysplasia, syndrome-associated conditions, a limb length discrepancy >1 cm, severe disorders of the feet, or pathologic growth. Figure 1 contains the study flowchart. Prior to inclusion, and due to the lack of previous data, we conducted a sensitivity-analysis using G*Power (v. 3.1.9.3, Faul and Buchner et al., Düsseldorf, Germany). To achieve a sensitivity of 0.8 and a moderate effect size of dz = 0.5 (Mann-Whitney U-Test), a population of *n* = 35 was calculated as being necessary. Biometric data were collected, along with a health-related quality of life assessment using the KINDL^®^ questionnaire, a validated instrument designed to evaluate physical, emotional, and social wellbeing in children. The questionnaire exists in two versions, KINDL^®^-Kid for younger children (7–13 years) and KINDL^®^-Kiddo for adolescents (14–17 years), as originally described by Ravens-Sieberer and colleagues in 1998 [19]. Motivational aspects of both children and their parents concerning potential surgery were evaluated using a custom-designed questionnaire, with all assessments conducted on the same day.

### Radiographs

Those patients who had undergone bilateral full-length standing radiography of the lower limb were recruited for our study during their routine visit in our outpatient clinic. To obtain true A.P. full-length radiographs, patients had to stand with both knees in a strictly forward position, where the leg rotation was adjusted so that the patella was centered medio-laterally and the X-ray beam was centered over the patella. Three radiographs were taken (hip, knee, and ankle) and then superimposed via an automated digital stitching technique. The radiographic leg alignment was automatically analyzed according to the latest gold standard in literature using artificial intelligence (Leg Angle Measurement Assistant LAMA, Image Biopsy Lab, Vienna, Austria). LAMA showed a mean absolute error of 0.30° for HKA angles and an ICC of up to 1.00, indicating excellent agreement with manual measurements [20–23].

### Video analysis

**Fig. 2:**
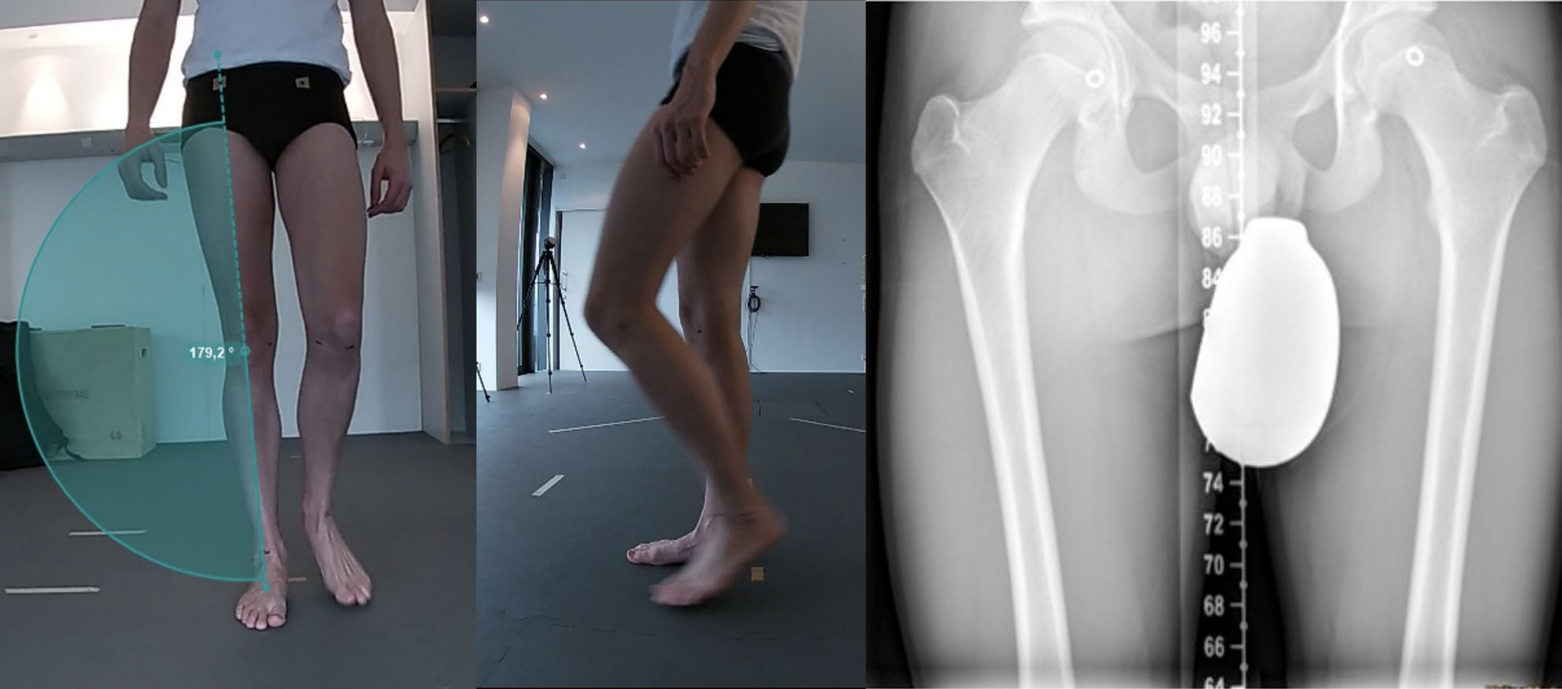
Mechanical leg axis/HKA determination based on anatomical landmarks, midstance gait analysis in two planes, and radiographic verification of the hip joint center

For gait analysis, participants walked four meters in a straight line, barefoot at their own comfortable speed, concurrently with the other biomechanical assessments. Videography was performed using commercially available high-speed video cameras with 1080 px resolution at a frame rate of 60fps. (GoPro Series Hero 7, 1080 pixels/60 fps). Cameras were placed in front of and orthogonally to a four-meter long walkway at a height of approx. 50 cm. A minimum of two trial runs were collected, and during the second trial, the subject was asked to stop and stand at the center of the camera’s field of view in the middle of the walkway to collect static data. Biomechanical analysis was performed using Kinovea software (Version 0.9.5, Kinovea, Bordeaux, France), an open access 2D movement-analysis program. Biomechanical analysis was performed independently by two investigators (A and B) to measure interrater reliability. Measurements were taken under dynamic (midstance gait, controlled in both planes via video) and static (bipedal stance) conditions. The leg axis was determined by marking anatomical landmarks: the hip joint center (clinically identified, marked with a circular metal marker, and verified or corrected on radiographs), as well as the knee joint center, medial and lateral joint lines, and the ankle joint center between the malleoli, which were identified clinically and marked on the skin. The primary investigator took all measurements twice in all subjects to determine intrarater reliability (Fig. 2).

### Statistical approach and outcomes

Primary outcome measures were any correlation between radiographic and video-based parameters of the coronal leg axis: hip-knee-ankle angle (HKA) and secondary parameters such as the medial proximal tibia angle (MPTA) and distal lateral femoral angle (LDFA) relying on reference values according to Paley et al. [3]. Our secondary outcome was the inter- and intrarater reliability of these measurements.

Our statistical approach followed a non-inferiority design. Statistical analysis was performed using SPSS v27 (IBM Corp., New York, USA). Continuous variables are reported as mean ± standard deviation, and discrete variables are reported as frequency (%). Data were first controlled for normal distribution using Shapiro-Wilk’s test. Mann-Whitney U test was used to determine differences between groups when data were not normally distributed. All p-values were two-sided with statistical significance defined at α = 0.05. Correlation analysis was performed using Pearson’s test if data were distributed normally, otherwise Spearman’s correlation coefficient was used. The correlation was interpreted following Cohen with 0.3–0.5 as weak, 0.5–0.7 as moderate and > 0.7 as a strong correlation. Intra- and Interrater-Reliability was calculated using the intraclass correlation coefficient.

## Results

### Study population

A total of 40 participants was included in our study, the mean age was 12.6 years (± 1.7). 45% were female, mean BMI was 22.94 kg/m^2^. In our population, 36 patients had genu valgum, while four patients had a genu varum; positive angles represent valgus. The mean HKA in radiographic analysis was 2.25° ± 3.55° (range − 14/+8). The video-based measurements revealed a smaller axis deviation in comparison to the X-ray values. Table 1 shows an overview of the data distribution and the standard measurement error for each modality.

**Table 1:** Descriptive statistics HKA (hip-knee-ankle-angle). Rx = radiograph, dyn. = dynamic, video-based measurement, stat. = static, video-based measurement, A = Investigator A, B = Investigator B., std. = standard

total *n* = 80	mean	std. error	std. deviation	min/max
HKA-Rx.	2.25	0.40	3.55	−14.6/8.0
HKA-dyn.-A	1.48	0.36	3.22	−17.8/5.8
HKA-stat.-A	1.79	0.35	3.11	−15.7/5.6
HKA-dyn.-B	0.93	0.42	3.77	−21.9/7.2
HKA-stat-B	1.69	0.34	3.07	−13.7/7.7

According to Shapiro-Wilk-Test HKA (*p* < 0.001), most of the secondary mMPTA and mLDFA angles of the knee were not normally distributed. The mean mMPTA-Rx in radiographic analysis was 88.14° ± 3.72° (71–93°). The video-based measurements were significantly different: mMPTA-dyn-A 88.00° ± 2.76, mMPTA-stat-A 88.52° ± 2.39°, mMPTA-dyn-B 85.56° ± 3.64°, mMPTA-stat-B 87.15° ± 2.97°.

The mean mLDFA-Rx was 85.56° ± 3.14° (74–93°). The video-based measurements were also significantly different: mLDFA-dyn-A 86.16° ± 2.76, mLDFA-stat-A 86.12° ± 2.76°, mLDFA-dyn-B 85.81° ± 2.98°, mLDFA-stat-B 86.79° ± 3.11°.

### Correlation analysis

There was a significant, positive correlation (*p* < 0.001) between all techniques (radiographic/dynamic/static). According to the nonparametric Spearman’s-Test and interpreted following Cohen, there were strong correlations across all groups (Table 2).

**Table 2:** Spearman’s correlation between radiography and video-based analysis, high correlation *r* > 0.5. HKA = hip-knee-ankle-angle, Rx = radiograph, dyn. = dynamic, video-based measurement, stat. = static, video-based measurement, A = Investigator A, B = Investigator B., std. = standard

	HKA-dyn.-A	HKA-stat.-A	HKA-dyn.-B	HKA-stat.-B
HKA-Rx.	0.868**	0.876**	0.545**	0.561**
HKA-dyn.-A	--	0.852**	0.718**	0.681**
HKA-stat.-A	--	--	0.603**	0.717**
HKA-dyn.-B	--	--	--	0.655**

**Fig. 3:**
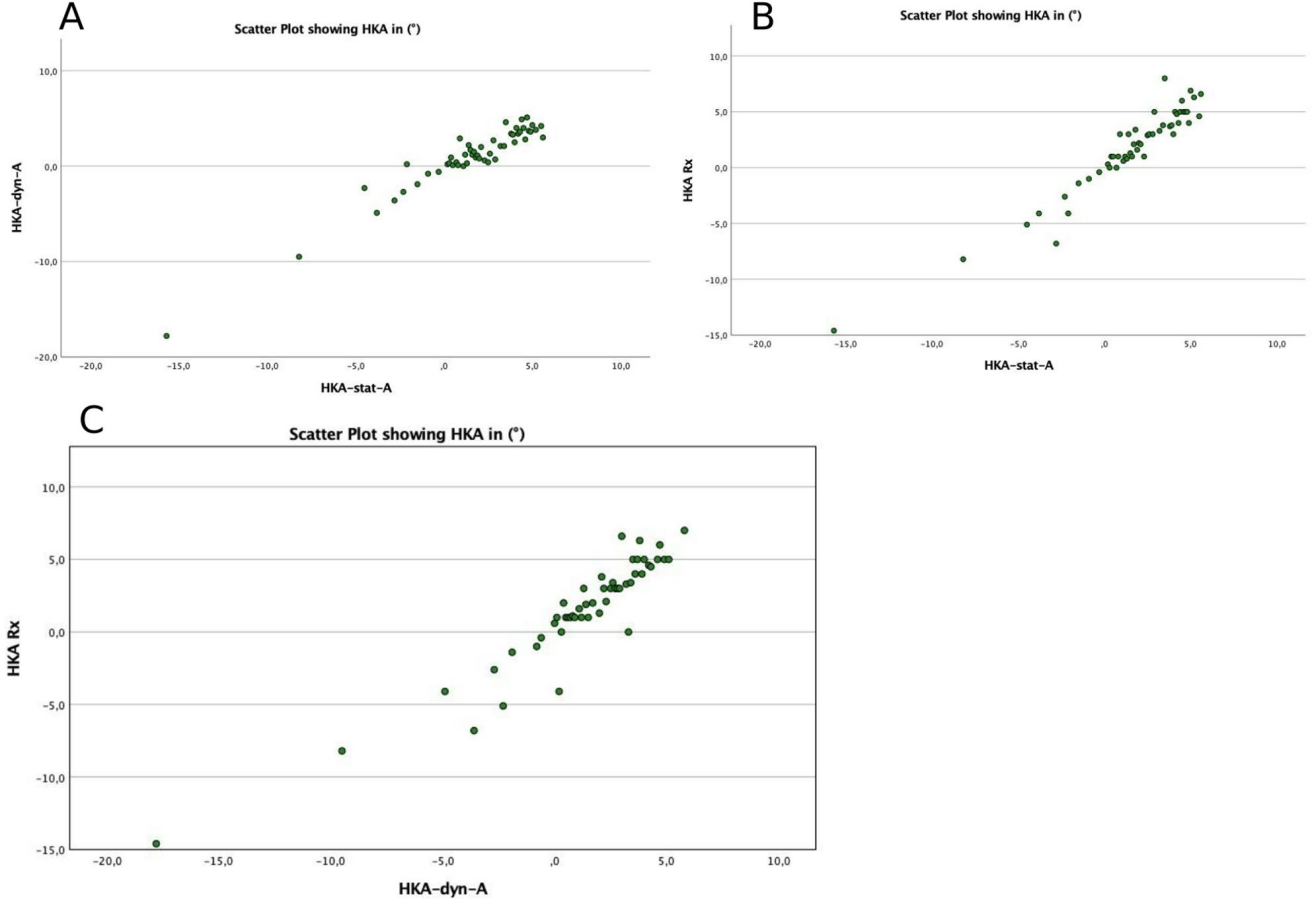
**A**: Correlation of dynamic and static measurement of HKA of investigator **A**. **B**: Correlation between HKA radiologically and static (Investigator **A**). **C**: Correlation between HKA radiologically and dynamic of investigator **A**

The scatter plots of the different measurement modalities are displayed in Fig. 3 investigator A and the radiological analysis.

There were significant correlations between radiographic and video-based analyses from investigator A: mMPTA-Rx/mMPTA-stat-A rho = 0.65**, mMPTA-Rx/mMPTA-dyn-A rho = 0.48**. This was also apparent in the mLDFA correlation analysis: mLDFA-Rx/mLDFA-stat-A rho = 0.73**, mLDFA-Rx/mLDFA-dyn-A rho = 0.75**.

**Fig. 4:**
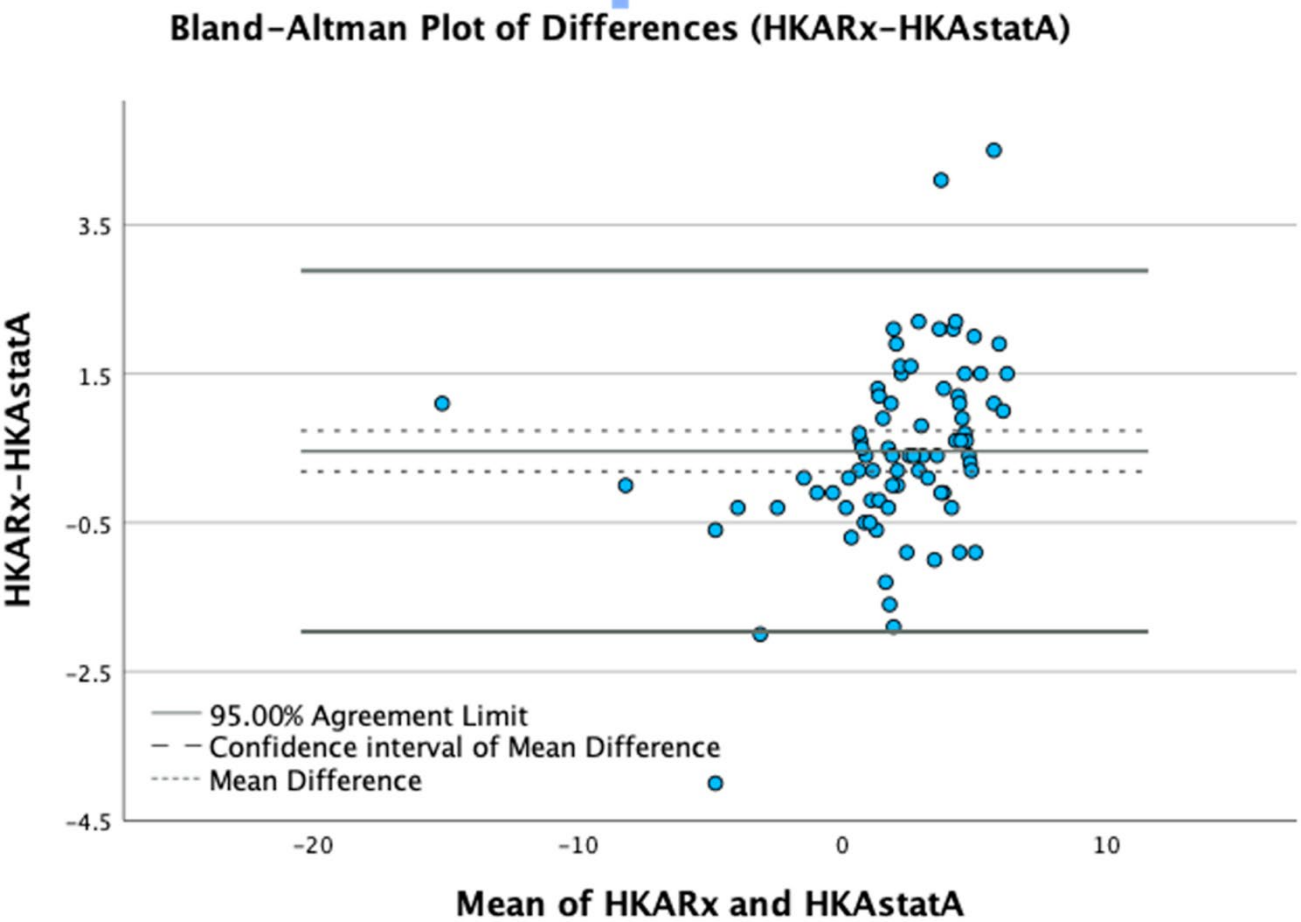
Bland–Altman plot of radiographic HKA (HKARx) versus static video-based HKA (HKAstatA). The mean difference is shown as a dotted line, with dashed lines indicating the confidence interval of the mean difference and solid lines the 95% limits of agreement

The Bland–Altman plot in Fig. 4 demonstrated a small mean difference between radiographic and video-based static HKA measurements, with the majority of values lying within the 95% limits of agreement, indicating good concordance between the two methods.

### Measurement reliability

Our HKA measurements showed high to excellent inter- and intrarater reliability. Regarding additional knee angles, interrater reliability was low for static parameters and dynamic parameters whereas intrarater reliability was high with ICC values > 0.7, see Table 3.

**Table 3:** ICC (intraclass correlation coefficient) values for Intra- and interrater reliability for knee angles

	HKA stat.	HKA dyn.	mMPTA-stat.	mMPTW-dyn.	mLDFA-stat.	mLDFW-dyn.
ICC- Interrater	0.821	0.874	0.437	0.492	0.295	0.286
ICC- Intrarater	0.993	0.994	0.774	0.881	0.925	0.832

### Questionnaires

**Fig. 5:**
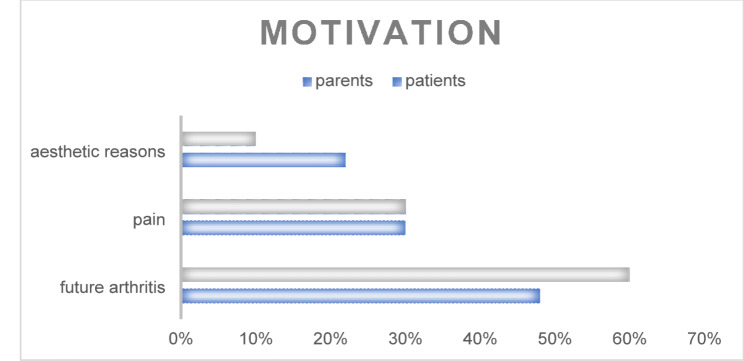
Motivation to undergo temporary surgical hemiepiphyseodesis

The main motivation in pediatric patients and their parents for seeking surgery was the hope of preventing osteoarthritis in adulthood. Aesthetic aspects were seldom part of the decision (Fig. 5).

## Discussion

In the present study, we evaluated frontal lower limb alignment in a pediatric population presenting with idiopathic genu valgum or varum. The main finding of this investigation was that the video-based static hip-knee-ankle-angle measurements correlate very closely with radiographic values, which is best demonstrated in Fig. 4. In our study, the static video-based measurements showed the higher correlation with radiographic measurements than the dynamic, video-based measurements, which is plausible as both methods assess the alignment under static conditions. Reliability assessments showed excellent inter- and intrarater reliability. Therefore, this low-budget technique should be considered a reliable alternative to traditional radiography for longitudinal evaluation of lower limb malalignment in children, especially when frequent radiographic follow-ups are necessary.

In our study, the static video-based measurements correlated best with radiographic measurements which makes sense because the radiograph is also a static method. Since surgical indication is based on radiographic assessment, static video analysis appears more suitable for follow-up monitoring, with radiographs recommended once deviations approach 2°, as these are also static measurements. However, video analysis alone is not sufficient for decision-making, as secondary knee angles could not be determined with adequate accuracy, and radiographs remain necessary to differentiate between tibial and femoral correction.

Applying this novel technique for the longitudinal analysis of lower limb alignment will save children from an otherwise considerable amount of ionizing radiation. In light of the measurement error of 0.34–0.42 in video-based analyses – compared to 0.40 in radiographic measurement, we suggest that a radiographic rather than video-based follow-up might only be necessary when the patient is approaching 1–2° of the target HKA. However, video analysis alone is not sufficient for decision-making, as secondary knee angles could not be determined with adequate accuracy, and radiographs remain necessary to differentiate between tibial and femoral correction.

It might be practicable to estimate the offset between the radiographic and video-based leg axis at the initial measurement and refer to that as a corrective factor for the potential deviation in future measurements. Strong correlations have also been reported when comparing motion-capture-systems to radiographic measurements [24, 25]. Other clinical methods such as inclino- or goniometry without radiation have demonstrated weaker correlations [26]. These methodologies, however, require much more infrastructure and may therefore not be suitable for broad application.

Relying on the findings of this study, video-based analysis seems inappropriate for calculating secondary knee angles. The measurement error and within-group deviation for these values are too large to apply them in a clinical or a research setting. A reason for this could be the habitus of the children and therefore greater difficulty in identifying the relevant anatomical structures, e.g. the joint line. We did not observe a correlation with BMI in our cohort, likely due to the limited sample size, although an influence seems plausible. Regarding skin color, all participants in this study had light skin, so potential effects of skin pigmentation on landmark detection could not be evaluated.

While the ICC values for the interrater assessment are not particularly strong, the intrarater ICCs are better, suggesting a systematic error in the angle measurements. Overall, alignment of the leg axis is fairly consistent, but the individual angles are not. When looking at the motivational background of a surgical decision, patients and parents seemingly follow the medical indication. Pain and early osteoarthritis due to severe leg axis deviation account for 75–90% of the motivational reasons. In the currently available data, aesthetic considerations do not play a primary role in the indication for temporary hemiepiphysiodesis from the perspective of healthcare providers, however, in our population, patients seem to be motivated by aesthetic concerns in 22% of the cases, the survey did not indicate evidence of psychological burden of the patients.

Furthermore, it was observed that in some cases, the dynamic and static measurements differed due to compensatory mechanisms during gait. Such effects are not accurately represented in static measurements. However, the clinical relevance of this dynamic deviation remains unclear. Farr et al. analyzed kinematic gait characteristics compared to radiography in children with genu valgum, which also showed a weak correlation between important radiographic parameters and frontal knee moments. This evidence indicates that rotational compensatory movements may occur that are not captured in radiographs [1]. The clinical importance of these compensatory mechanisms and the discrepancy between static and dynamic angles require further analysis. Phenomena derived from an adult population, such as valgus/varus thrust, have not been investigated in children. However, transient hemiephiphysiodesis is a three-dimensional correction that also has an effect on rotational and dynamic components. To date, such data are not sufficiently acknowledged in clinical decision-making or when determining the exact placement of the implants. In part, this is attributable to the fact that the dynamic analysis of leg alignment remains challenging. Future studies will therefore need to assess this potential with regard to rotational and dynamic malalignment to further improve such “guided growth” treatment. From our findings, however, we must conclude that the current methodology of video-based analysis is not yet accurate enough for analyzing rotational components and additional knee angles.

### Limitations

This study’s limitations include its relatively small sample size and the inhomogeneity between varus and valgus knees. The hip center was marked according to clinical examination evidence and thus depends on the experience of the clinical investigator and the patient’s habitus. Sonographic measurements might prove useful to improve measurement accuracy [27]. This study’s cohort was also too small to investigate the influence of BMI on analysis accuracy. There is some evidence that BMI has a significant influence on the accuracy of video-based gait analysis [24]. Future studies should evaluate different techniques to identify anatomical landmarks, investigate a larger cohort, and evaluate the reliability of video-based lower limb axis analysis during gait in a longitudinal study protocol.

## Conclusion

In summary, current popular techniques to enable “guided growth” aim to correct frontal plane deformities by the transient fixation of one side of the epiphysis. Indications for this approach derive mostly from static radiography. This study shows that video-based measures correlate closely with radiographic measures for the HKA, whereas the current methodology of video-based analysis failed to be sufficiently accurate for analyzing rotational components and additional knee angles. Future studies should therefore investigate whether video-based lower-limb analysis is useful for monitoring changes in the lower limb axis during guided growth, thereby minimizing the use of radiation in pediatric orthopedics.

## Supplementary Information

Supplementary Material 1.

## Data Availability

The data that support the findings of this study are not openly available due to reasons of sensitivity. They are available from the corresponding author upon reasonable request. Data are located in controlled access data storage at University of Freiburg.

## Abbreviations

BMI: Body Mass Index
DRKS: Deutsches Register Klinischer Studien (German Registry of Clinical Studies)
HKA: Hip–Knee–Ankle Angle
ICC: Intraclass Correlation Coefficient
LDFA: Lateral Distal Femoral Angle
LAMA: Leg Angle Measurement Assistant
MPTA: Medial Proximal Tibial Angle
Rx: Radiograph
SPSS: Statistical Package for the Social Sciences
