# Supplementary material for: Prospective video-based analysis of coronal lower limb alignment may be as accurate as radiography in children
Source: BMC Musculoskelet Disord. 2025 Nov 27;26:1073. doi: 10.1186/s12891-025-09343-y (PMC12659304; doi:10.1186/s12891-025-09343-y)
Supplement: Supplementary file 1 — Supplementary Material 1. [file 12891_2025_9343_MOESM1_ESM.pdf]

Questionnaire on Symptoms and Surgery related to Pathological Lower Limb Alignment

Dear patient, dear parents,

Please fill out the following questionnaire. If you have any questions, feel free to ask during your outpatient visit.

Thank you for your cooperation!

1. **Which deviation from physiological leg alignment is present?**

   ☐ Genu varum/bow legs ☐ Genu valgum/knock knees

2. **Patient: Can you imagine undergoing surgery to reduce or correct your leg alignment?**

   ☐ yes ☐ no

3. **Patient: I am bothered by my bow legs / knock knees because...**

   ☐ I don't like the appearance / I find it unattractive

   ☐ Other people notice it

   ☐ I have pain

   ☐ I am afraid it might harm my knee in the long term

5. **Parents: Would you consider surgery to correct your child's leg alignment?**

   ☐ yes ☐ no

6. **Parents: I would consider surgery to improve my child's leg alignment...**

   ☐ for aesthetic reasons

   ☐ because my child has complaints

   ☐ to prevent osteoarthritis in the long term
